# Words of Consolation

**Published:** 1886-10-09

**Authors:** 


### WORDS OF CONSOLATION.

Communications for this column should be addressed, "Chaplain," care of the Editor of THE HOSPITAL.

II.

In contemplating the religious aspects of sickness, it may be stated broadly that there are two classes of sufferers, those who suffer as Christians, and those who suffer as ordinary mortals, because they must. If we would suffer as Christians, there is no lack of assurance in Holy Scripture that sickness may be changed from a seeming curse to the greatest of blessings. But however difficult this may be to discern when we are first called to suffer, we may, at all events, begin well by simply believing that God has a merciful and loving purpose in all that He ordains for us to bear. Sufferers cannot generally be supposed to see what this purpose is all at once. It sometimes takes very long before God's meaning in His chastisements is discerned by His children; and those who try to point out the reasons for His chastening are often too hasty in their conclusions, and so do more to perplex than to comfort a sufferer. No; in the majority of cases, those who would suffer even as Christians do not at once see the "why" or "wherefor" of their affliction. Some are given to understand the Divine intention at a very early stage in sickness; they are happy in their discernment; and they become very patient and resigned in consequence. But many must be quite content at first by believing that God *has* a purpose, although they have to await the explanation, leaving Him to unfold the secret of His visitation. It is surely a comfort to be able from one's heart to believe as much as this. "God has a purpose in my sufferings, and because He is God it must be a good purpose, it must be a wise purpose, it must be a loving purpose."

Sickness has its own temptations and spiritual trials, which those who are in health know nothing about. One of these is to fancy, because we are laid by, whether it be for a long or short period, that the time spent in hospital must be so much waste for ourselves, or so much loss to those who depend on us. Such an idea will not be entertained for more than a moment by one who believes that God has a purpose in sending sickness, quite as distinct in its outline and plan as when He gave health and strength for much labour or enjoyment of life. It may be your lot as a sufferer to be content with this thought for awhile, without seeing any further. Meanwhile you may pray, "Show me Thy ways, O Lord; and teach me Thy paths. Lead me forth in Thy truth, and learn me; for Thou art the God of my salvation; in Thee hath been my hope all the day long" (Psalm xxv, 3-4).

Then you may simply rest in the Lord's hands without giving way to fretfulness or over-anxiety about yourself or about your family; for "He doth not afflict willingly, nor grieve the children of men" (Sam. iii, 33).

